# Comparative effectiveness of dual-focus contact lenses and DIMS spectacle lenses for myopia control in patients with low astigmatism: a retrospective study

**DOI:** 10.3389/fmed.2025.1524700

**Published:** 2025-06-18

**Authors:** Chia-Yi Lee, Shun-Fa Yang, Yu-Ling Chang, Jing-Yang Huang, Ie-Bin Lian, Chao-Kai Chang

**Affiliations:** ^1^Institute of Medicine, Chung Shan Medical University, Taichung, Taiwan; ^2^Nobel Eye Institute, Taipei, Taiwan; ^3^Department of Ophthalmology, Jen-Ai Hospital Dali Branch, Taichung, Taiwan; ^4^Department of Medical Research, Chung Shan Medical University Hospital, Taichung, Taiwan; ^5^Department of Medical Education, Cathay General Hospital, Taipei, Taiwan; ^6^Institute of Statistical and Information Science, National Changhua University of Education, Changhua, Taiwan; ^7^Department of Optometry, Yuanpei University of Medical Technology, Hsinchu, Taiwan

**Keywords:** defocus incorporated multiple segments’, dual-focus contact lenses, axial length, spherical equivalent refraction, astigmatism

## Abstract

**Purpose:**

This study aimed to evaluate and compare the effectiveness of dual-focus contact lenses (DFCL) and defocus incorporated multiple segment (DIMS) spectacle lenses in controlling myopia progression in patients with low astigmatism.

**Methods:**

A retrospective cohort study was conducted involving myopic patients with astigmatism of less than −1.25 diopters (D) who used either DFCL or DIMS spectacle lenses. The study included 95 eyes in the DFCL group and 88 eyes in the DIMS group, and the spherical equivalent refraction (SER) of the study population ranged from −0.50 D to −6.00 D. The primary outcomes were the progression of SER and the elongation of axial length (AXL) over 1 year. A generalized linear mixed model was used to calculate adjusted odds ratios (aORs) and the associated 95% confidence intervals (CIs), adjusting for age, sex, initial AXL, and initial SER.

**Results:**

The mean SER progression was −0.28 ± 0.15 D in the DFCL group and −0.25 ± 0.12 D in the DIMS group (*p* = 0.139). The mean AXL elongation was 0.12 ± 0.07 mm for DFCL users and 0.10 ± 0.05 mm for DIMS users (*p* = 0.029). Trend analysis revealed no significant differences in SER progression (aOR: 0.988; 95% CI: 0.945–1.033; *p* = 0.513) or AXL elongation (aOR: 0.982; 95% CI: 0.945–1.018; *p* = 0.307) between the groups after adjusting for confounders. Subgroup analyses indicated no significant differences in SER progression or AXL elongation between DIMS and DFCL users across different baseline characteristics (all *p* > 0.05).

**Conclusion:**

The use of DIMS spectacle lenses showed SER and AXL control similar to that of DFCL.

## 1 Introduction

Myopia is a disease with an increasing rate of prevalence in recent times, characterized by blurry vision at a far distance (1, 2). The incidence of myopia development is above 40%/year in the American population (3) and more than 80%/year in the Eastern Asian region due to the differences in lifestyle and education (3). The mechanism of myopia formation is the steepening of corneal curvature and eyeball elongation, which tend to persist before 18 years of age (4). The late onset of myopia is primarily a result of the impairment of the accommodative stimulus/response functions (5). High myopia, defined as a spherical equivalent refraction (SER) of more than −6.00 diopters (D) (6), may elevate the risk of retinal detachment, optic nerve damage, and myopic maculopathy; thus, retardation of high myopia development is warranted in current situation (7).

Different methods have been developed to control myopia in the past decades (1, 8). Instilling high-concentration atropine (ATR) is most effective in reducing myopia progression, while complications such as ocular allergy, photophobia, and blurry vision at reading distance could develop and reduce compliance (2, 9). The orthokeratology contact lens can improve myopia control regarding refraction (10, 11). Almost all individuals with orthokeratology contact lenses benefit from spectacle independence (12). Comparing the efficiency of ATR usage and orthokeratology contact lens wear on controlling myopia, the rate of the SER progression and the axial length (AXL) elongation demonstrated analogous results between the high concentration ATR and the orthokeratology contact lens (13). In a review article, the orthokeratology contact lens provides slightly less than 50% of myopic control, while the high-dose ATR presents a 73% effect on myopia control (2).

A certain new myopia-control method has been introduced recently (14). Most of these methods applied the multifocal lenses with through-focus characteristics, which incorporate several radial or angular zones with different refractive powers or low- and high-order aberration combinations, and the 3–4 zone design leads to the best through-focus performance (15). The peak performance and range over threshold through-focus vary among different refractive multifocal designs according to the analysis that integrates Fourier optics and Zernike-based wavefronts (16). In addition to the design itself, the orientation of multifocal lens designs with angular addition increments would also affect the optical performance of multifocal lenses (17). The dual-focus contact lenses (DFCLs) were a soft contact lens that was applied in the past decade, which contributed to acceptable myopic control of the SER progression and AXL elongation compared to patients with simple soft contact lenses (14). Besides, the defocus incorporated multiple segments (DIMS) spectacle lenses were also applied recently, with adequate myopic control efficiency concerning AXL elongation (18, 19), and the independence of contact lens usage of DIMS spectacle lens preserves the health of the ocular surface (20). About the efficiency among different myopic control interventions, a previous review article suggested the ATR, orthokeratology contact lens, and multifocal soft contact lenses may control the AXL elongation, while the results of peripheral plus spectacles on myopic control were inconsistent (21). Another study enrolling the results of recent randomized control trials demonstrated that orthokeratology contact lens, DFCL and DIMS are effective in myopic control while the low-dose ATR was minimal and effective in retarding AXL progression in two articles (22). Nevertheless, there were limited studies that evaluated the myopic control efficiency of the DFCL and DIMS spectacle lens. Different designs of the DFCL and DIMS spectacle lenses may result in variations in myopic control (14, 18).

Consequently, the present study aims to evaluate the myopic control effect between DFCL and DIMS spectacle lenses regarding the SER progression and AXL elongation.

## 2 Materials and methods

### 2.1 Inclusion criteria

The retrospective cohort study was conducted at Nobel Eye Institute, a joint clinic group with several branches in the northern, southern, and central fields of Taiwan. Inclusion criteria of participants in the present study including: (1) age from 8 to 15 years-old, (2) apply either DFCL or DIMS spectacle lens in our clinics during 1 January 2021 to 31 December 2022, (3) astigmatism lower than −1.25 D, and (4) regularly followed up in any branch of Nobel Eye Institute for more than 1 year. Only patients with low astigmatism were enrolled in the present study because DFCL lacks astigmatic correction function and is not indicated for high astigmatism. The present study utilized one brand of DFCL (MiSight, CooperVision, Victor, NY, US) and one DIMS spectacle lens (Miyosmart, Hoya, Shinjuku-ku, Tokyo, Japan).

### 2.2 Exclusion criteria

To enhance the homogeneity of the study population, we adopted the following exclusion criteria: (a) best-corrected visual acuity (BCVA) cannot reach 20/40 at a Snellen chart at the first visit; (b) high myopia, that is, SER greater than −6.00 D (6); (c) usage of any ATR eyedrop; (d) severe eye diseases, including but not limited to corneal neovascularization, corneal scar, infantile glaucoma, congenital cataract, significant retinopathy of prematurity, retinal detachment, optic nerve damage, and achromatopsia. Only one eye of each participant was randomly selected for the present study, and whether to include the right or left eye was decided via drawing lots. After the whole selection and exclusion action, a total of 95 and 88 eyes were taken to the DFCL group and the DIMS group after the whole selection process, respectively.

### 2.3 Usage of myopia control interventions

Concerning the usage of DFCL and DIMS, patients who used DFCL were suggested to continuously wear DFCL for approximately 10–12 h per day. After discontinuing DFCL, they will use single vision spectacles to gain acceptable vision for the rest of the day. Furthermore, patients were advised to remove DFCL 3–4 h before the refractometry and other exams in each visit. Since the previous publications regarding DFCL did not propose a removal-to-measurement time (14, 23) We decided on this time interval based on our experience. Conversely, the patients receiving DIMS management were suggested to wear it for at least 8 h per day, and they can keep wearing the DIMS for the rest of the time if there is no need (i.e., swimming, basketball game) to remove the glasses. They are allowed to take off DIMS while exercising or napping; thus, the total time of usage is shorter than that of the DFCL.

### 2.4 Major outcome

The baseline features of these participants, including age, sex, pre-management BCVA, manifest/cycloplegia sphere power, manifest/cycloplegia cylinder power, and AXL, were taken from our documents. About myopic progression parameters, the major outcomes in the present study are manifest/cycloplegia SER progression and AXL elongation after the 1-year study interval. The manifest SER as well as AXL were evaluated by an autorefractor (Brand: KR-8900, Topcon, Itabashi-ku, Tokyo, Japan) and a biometry device (Brand: IOL Master 500, Carl Zeiss, Göschwitzer Str., Jena, Germany) in each branch of Nobel Eye Institute. About the measurement of cycloplegia SER, a topical cycloplegic agent, tropicamide (Better eye drop, Aseptic Innovative Medicine Co. Ltd., Taoyuan dist., Taoyuan, Taiwan) was instilled about two to four times before the measurement. The optometrists checked the pupil diameter, and refraction measurement would be done if the pupil diameter was wider than 8 mm. The manifest and cycloplegia refractions include both sphere and cylinder powers, which were calibrated three times according to the instructions received from the manufacturer. Then, the average value of the three measurements was obtained, and sphere power plus 50% of cylinder power was regarded as manifest and cycloplegia SER in the present study. Both manifest SER and AXL values before myopic treatment, 3 months after myopic treatment, 6 months after myopic treatment, 9 months after myopic treatment, and 12 months after myopic treatment in the DFCL and DIMS groups were analyzed in the subsequent analysis. On the contrary, cycloplegia SER before myopic treatment and 12 months after myopic treatment in the DFCL and DIMS groups were analyzed.

### 2.5 Statistical analysis

The SPSS 20.0 version (Brand: SPSS Inc., Chicago, Illinois, USA) was utilized for statistical analyses of the present study. The Shapiro–Wilk test was used to confirm the normality of the data in our study population (all *p* > 0.05). Besides, the statistical power was 0.95 with a 0.05 alpha value and a medium effect size produced by G∗power version 3.1.9.2 (Heinrich-Heine-University, Düsseldorf, Germany). The number (N) in the text and tables of this study represents the number of eyes. A descriptive analysis was performed to show the baseline features of the DFCL and DIMS groups. Fisher’s exact test and independent t-test were utilized to compare baseline features between the two groups based on the character of the parameters. An independent t-test was subsequently conducted to compare baseline cycloplegia SER and AXL, final cycloplegia SER and AXL, as well as the increment in cycloplegia SER and AXL between the DFCL and DIMS groups. The generalized linear mixed model was used to evaluate the trend in manifest SER progression and the AXL elongation between DFCL and DIMS groups, with adaptation to the impact of age, sex, pre-management AXL, and pre-management SER. The adjusted odds ratio (aOR) plus 95% confidence interval (CI) of the DIMS group compared to the DFCL group was calculated using a generalized linear mixed model. The trends of manifest SER progression and the AXL elongation were presented on a line chart, with the standard error for both groups represented as error bars. Moreover, the generalized linear mixed model was applied to evaluate the correlation between SER progression (as an absolute value) and AXL elongation in the two groups. In subgroup analysis, the cycloplegia SER and AXL increments of participants who received DFCL or DIMS spectacle lenses with moderate myopia (more than −3.00 D after cycloplegia), high AXL (more than 25.00 mm), and young age (younger than 10 years old) were analyzed using the generalized linear mixed model. In this study, the statistical significance was defined as *p* < 0.05.

## 3 Results

The baseline features of the DFCL and DIMS groups are shown in Table 1. The mean age showed 10.78 ± 2.05 years in the DFCL group, which was non-significantly older than the DIMS group (10.49 ± 1.97 years, *p* = 0.331). The rest of the demographic data, including sex, laterality, pre-treatment BCVA, pre-treatment sphere power, and pre-treatment cylinder power, also did not illustrate a significant difference between the two groups (all *p* > 0.05) (Table 1).

**Table 1:** Basic characters between the two groups.

Character	DFCL group (*N* = 95)	DIMS group (*N* = 88)	*p*-value
Age	10.78 ± 2.05	10.49 ± 1.97	0.331
Sex (male:female)	42:53	43:45	0.315
Laterality (right:left)	49:46	42:46	0.355
Pre-treatment BCVA (LogMAR)	0.01 ± 0.03	0.01 ± 0.04	0.999
Pre-treatment sphere (D)	−2.46 ± 1.38	−2.40 ± 1.14	0.750
Pre-treatment cylinder (D)	−0.70 ± 0.22	−0.66 ± 0.20	0.201
Pre-treatment manifest SER (D)	−3.26 ± 1.44	−3.18 ± 1.19	0.684

AXL, axial length; D, diopter; DFCL, dual-focus contact lenses; DIMS, defocus incorporated multiple segments’; N, number; SER, spherical equivalent refraction.

**Figure 1:**
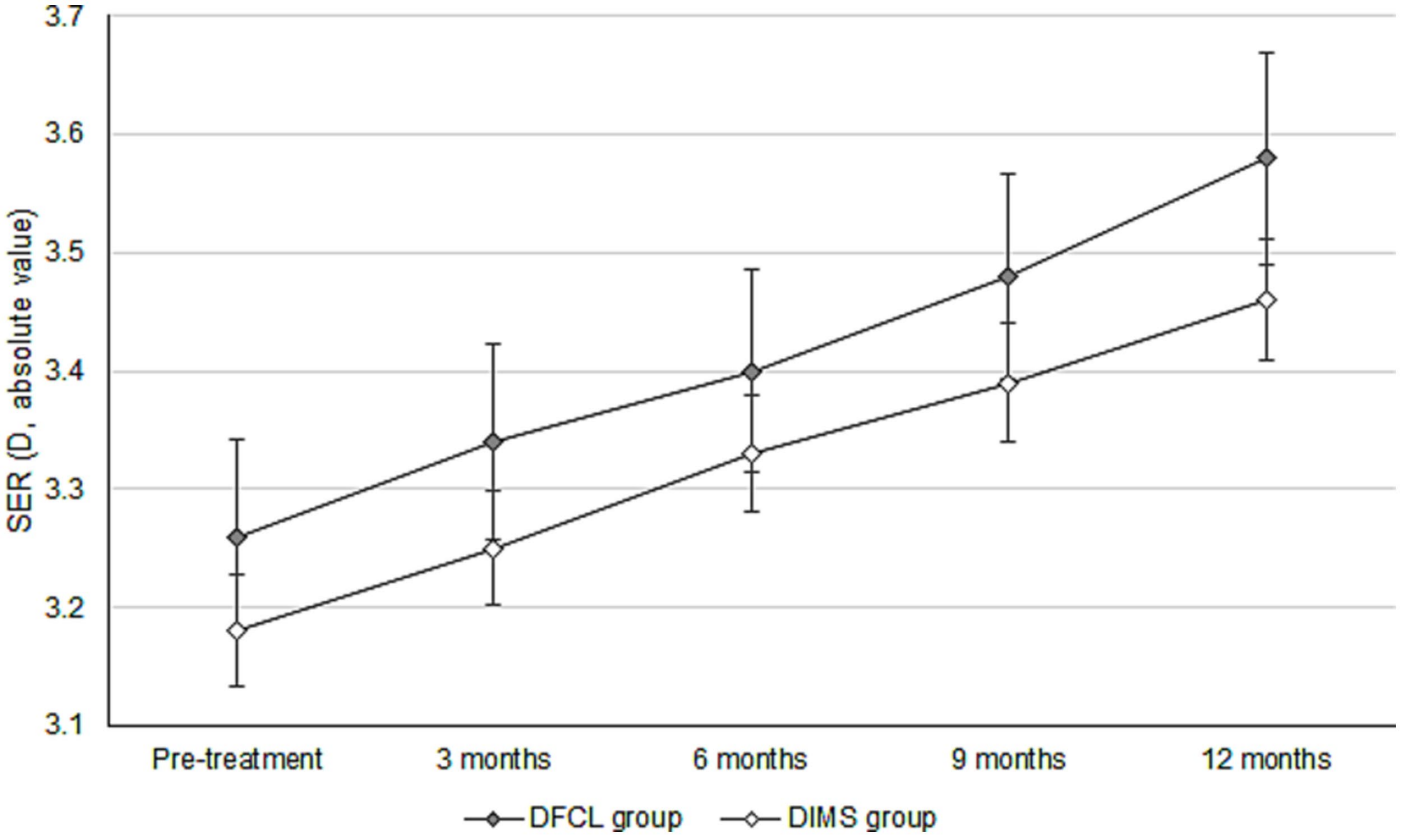
Trend of manifest spherical equivalent refraction change between the two groups. D, diopter, SER, spherical equivalent refraction, DFCL: dual-focus contact lenses, DIMS: defocus incorporated multiple segment.

**Figure 2:**
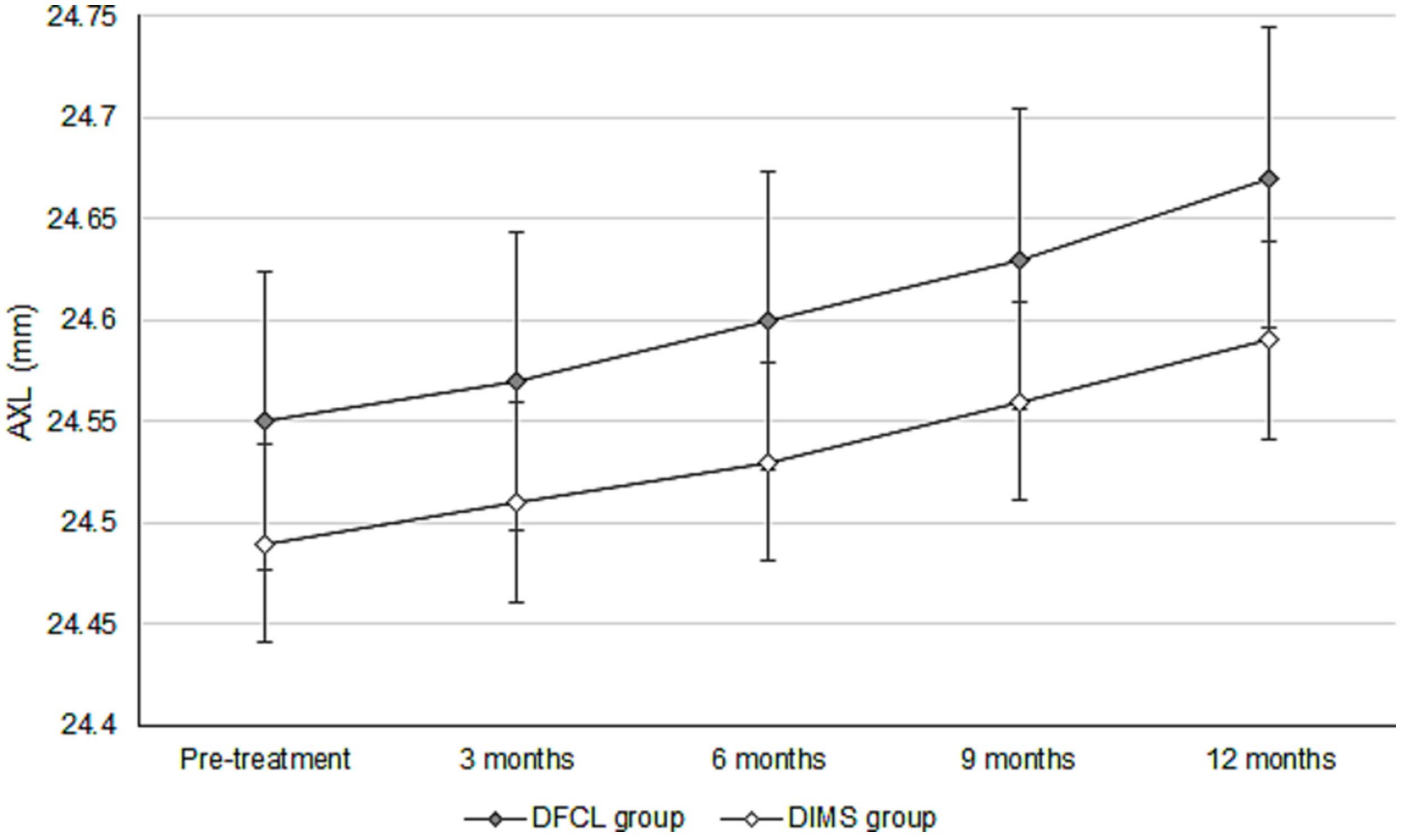
Trend of axial length changes between those groups. AXL, axial length, DFCL: dual-focus contact lenses, DIMS: defocusincorporated multiple segment.

The initial cycloplegia SER was −2.81 ± 1.32 D and −2.73 ± 1.10 D in the DFCL and DIMS groups, with no significant difference (*p* = 0.658). After the 1-year course of myopic management, the increase in cycloplegia SER was −0.28 ± 0.15 D in the DFCL group, which was similar to the −0.25 ± 0.12 D in the DIMS group (*p* = 0.139). Besides, the initial AXL was 24.55 ± 0.92 mm and 24.49 ± 0.93 mm in the DFCL and DIMS groups, which were also similar (*p* = 0.662). After the whole therapeutic interval, the increment of AXL was 0.12 ± 0.07 mm in the DFCL group and 0.10 ± 0.05 mm in the DIMS group, where the AXL elongations were significantly lower in the DIMS group (*p* = 0.029) (Table 2). For the trend analysis that adjusted several confounders, the DIMS group presented with a non-significantly lower trend of manifest SER progression compared to the DFCL group (aOR: 0.988, 95% CI: 0.945–1.033, *p* = 0.513) (Figure 1). On the other hand, the DIMS group also demonstrated a non-significantly lower trend of AXL elongation compared to the DFCL group (aOR: 0.982, 95% CI: 0.945–1.018, *p* = 0.307) (Figure 2). Besides, the change of SER (in absolute value) is positive correlated with the change of AXL in both the DFCL group (aOR: 1.663, 95% CI: 1.368–1.958, *p* < 0.001) and the DIMS group (aOR: 1.789, 95% CI: 1.478–2.100, *p* < 0.001).

**Table 2:** Change in cycloplegia spherical equivalent refraction and axial length between the two groups after the follow-up period.

Outcome	DFCL group	DIMS group	*p*-value
SER (D)			
Pre-treatment	−2.81 ± 1.32	−2.73 ± 1.10	0.658
Post-treatment	−3.09 ± 1.36	−2.98 ± 1.18	0.561
Increment	−0.28 ± 0.15	−0.25 ± 0.12	0.139
AXL (mm)			
Pre-treatment	24.55 ± 0.92	24.49 ± 0.93	0.662
Post-treatment	24.67 ± 0.95	24.59 ± 0.98	0.576
Increment	0.12 ± 0.07	0.10 ± 0.05	0.029*

AXL, axial length; DFCL, dual-focus contact lenses; D, diopter; DIMS, defocus incorporated multiple segments; SE, spherical equivalent refraction. *significant difference between the groups.

In the subgroup analysis, all the baseline characteristics between DFCL and DIMS subgroups in the same category did not show significant differences (all *p* > 0.05). All the DIMS subgroup of different characters presented with a similar value of SER progression compared to the DFCL group with the same conditions (all *p* > 0.05) (Table 3). Concerning the increment of AXL in different DFCL and DIMS sub-groups, the DIMS subgroups that featured with moderate myopia, high AXL, or young age also demonstrated an insignificantly lower rate of AXL elongation than the DFCL sub-groups with the same characteristics (all *p* > 0.05) (Table 3).

**Table 3:** Subgroup analysis between the two groups with different characteristics.

Subgroup	aOR^#^	95%CI	*p*-value
SER increment			
Moderate myopia	0.983	0.919–1.047	0.497
High AXL	0.975	0.937–1.014	0.202
Young age	0.996	0.953–1.039	0.924
AXL increment			
Moderate myopia	0.977	0.932–1.023	0.259
High AXL	0.968	0.936–1.000	0.053
Young age	0.991	0.948–1.035	0.878

AXL, axial length; aOR, adjusted odds ratio; CI, confidence interval; SER, spherical equivalent refraction. ^#^DIMS group toward DFCL group.

## 4 Discussion

In the present study, the tendencies for cycloplegia SER progression and AXL elongation were similar between the DIMS and DFCL groups according to multivariable analysis. Conversely, the value of AXL elongation was greater in the DFCL group than in the DIMS group. The subgroup analysis demonstrated similar risk of SER progression and AXL elongation between the DFCL and DIMS spectacle lens populations with different baseline characteristics.

The DFCL application showed a similar control on cycloplegia SER progression to the DIMS application, while the DIMS spectacle lens utilization presented with a lower AXL elongation than the DFCL application. In the previous research, the application of DFCL is associated with a significantly lower rate of SER progression and AXL elongation compared to those with single contact lens wear, whether in a 3-year or 6-year follow-up interval (14, 23). However, the application of DIMS spectacle lens demonstrated significant efficiency in myopia control regarding SER progression and AXL elongation compared to the patients who wear single vision spectacle lenses after 2-year treatment (18). Nevertheless, the previous studies only compared the DFCL or DIMS users to those without myopic control tool usage (i.e., ATR and orthokeratology contact lens), in which only the daily disposable contact lens or single vision spectacle was applied in the control groups of previous studies (14, 18, 23). No studies compared the efficiency of myopic control between DFCL and DIMS spectacle lenses in a population with the same ethnicity and living in the same region. The present study might be a preliminary experience demonstrating the difference in myopic control between DFCL and DIMS spectacle lenses in an Asian population. Furthermore, the individuals with prominent astigmatism higher than −1.25 D were excluded from the present study. Since the DFCL design cannot correct or reduce astigmatism, this exclusion process can remove some children who were not suitable for the DFCL application, but still used the DFCL due to their ambition (24). Besides, we adjusted several confounders of myopic control, such as age, initial sphere power, and initial AXL, in the generalized linear mixed model (4, 25, 26). Thus, the difference between the DFCL DIMS spectacle lens and myopic progression may be independent. Although the flattening effect on the corneal curvature existed in both soft contact lenses and orthokeratology contact lenses (27, 28), the corneal astigmatism did not reveal significant changes 1 year after the DFCL application. Thus, the cycloplegia SER measurement in the DFCL group may not be interfered with significantly by the flattening effect of the contact lens. The better AXL elongation control with DIMS spectacle lenses, on the other hand, could be due to the multifocal design of the DIMS spectacle lens, which can project the image on the retina with higher accuracy, and the retinal image clarity is an important risk factor for AXL elongation (20, 29). Still, the increment of AXL in the two groups was below 0.20 mm, which is an acceptable AXL control effect, and the difference of AXL elongation between the two groups was only 0.02 mm. Furthermore, the trends of SER and AXL changes did not illustrate a significant difference between the DFCL and DIMS groups. Consequently, the difference in AXL elongation between the DFCL and DIMS groups may not be clinically significant. Considering the daily wearing time, the patients with DFCL were suggested to wear for at least 12 h, and those with DIMS were suggested to wear for at least 8 h. According to our protocol, the DIMS may show similar myopic control efficiency as DFCL but with fewer hours of daily wearing. However, we told the patients that they could keep wearing DIMS all day if there was no need to remove them, and the average time of spectacle wearing in Taiwanese children was approximately 10–12 h, based on our clinical experience. Consequently, the daily wearing time between the DFCL and DIMS groups in the present study may be similar.

In subgroup analyses, the cycloplegia SER progression was similar in DFCL patients with moderate myopia, long AXL, and young age compared to the DIMS participants with the same conditions. According to previous publications, moderate myopia and high AXL could indicate higher difficulty in myopia control (30, 31). As a consequence, the SER progression in such a population may be more prominent in both the patients who received DIMS spectacle lens management and DFCL management, but the difference between the DFCL and DIMS groups did not illustrate a significant difference, which indicates the same efficiency of the two myopic control tools in high-risk populations. Moreover, the SER progression rate in individuals younger than 10 did not illustrate a significant difference between the DFCL and DIMS populations in the present study. Although the cycloplegia SER progression between the DFCL and DIMS groups was similar, the high convenience of DIMS spectacle lens utilization compared to the DFCL application might contribute to higher patient compliance and satisfaction in the younger population. Furthermore, the Asian population has smaller eyeball parameters than the Caucasian population (32). Thus, the DFCL usage in the crowded ocular surface of Asian children, especially for those younger individuals, might trigger some discomfort. On the other hand, the elongation of AXL is by far the most credible index for the myopic progression (33). Regarding the AXL-related analysis, the moderate myopia, high AXL, and young age subgroups that received DIMS spectacle lens management featured a similar AXL elongation control compared to the DFCL subgroups. There may have been a rare study to demonstrate this phenomenon before. These results, combined with the results of cycloplegia SER-associated subgroup analysis, may indicate that the DIMS spectacle lens controls myopia with similar efficiency in all children compared to the DFCL, and the absence of ocular surface involvement of DIMS may elevate the will of both parents and children to use DIMS since contact lens-related keratitis may develop (34). Further research is necessary to elucidate the efficiency of DFCL and DIMS usage.

Concerning the efficiency of myopic control in the present study and the previous literature, a preceding research revealed a SER progression and the AXL elongation of −0.51D and 0.30 mm in DFCL users, respectively, after 3 years (14). Besides, the extension of that research demonstrated a SER progression and the AXL elongation of −0.52D and 0.23 mm in DFCL users, respectively, after 6 years (23). The SER progression and the AXL elongation 1 year after the DFCL treatment were −0.28 D and 0.12 mm in the present study, which is comparable to the previous literature (14, 23). The previous study for DIMS spectacle lens showed a SER progression and an AXL elongation of −0.41 D and 0.21 mm after 2 years of follow-up and −0.50 D and 0.31 mm in the population who applied the DIMS spectacle lens for 3 years (18, 19). The change of cycloplegia SER in the present study was grossly similar to the previous experiences (18, 19), and the similar rate of AXL elongation may further indicate that the effect of myopic control between the present study and previous publications was similar, since the change of AXL is an accuracy index for the myopic progression (33).

All demographic data between the DFCL and DIMS groups were similar. The age was numerically higher in the DFCL group than in the DIMS group. This difference may be reasonable since the applicable age of DFCL in Taiwan was set at 9 years according to the consensus of most ophthalmologists and the health bureau. Consequently, younger children could choose the DIMS spectacle lens as the myopic control method. However, the mean age difference between the DFCL and DIMS groups was only 0.29 years, which means approximately 3.5 months and may not significantly influence myopic progression. Furthermore, we adjusted the effect of age on myopic control in the generalized linear mixed model; thus, the influence of different ages on myopic progression may not be prominent (35, 36).

There are still some limitations to the present study. First, the retrospective design of the present study would decrease the homogeneity of this myopia population compared to a prospective one, although we excluded some extreme conditions. Second, the non-randomized assignment to DIMS or DFCL due to the patients’ autonomy and the retrospective design of the present study could contribute to biases that interfere with the treatment choice and the study results. In addition, we did not perform cycloplegia refractions on each patient at each visit due to the parents’ compliance with clinical practice. Although we collected the cycloplegia SER before and 1 year after the DFCL and DIMS applications, the lack of trend analysis could prominently influence the integrity of the myopic control analysis. Moreover, the study population only consisted of 183 eyes from 183 participants, and such small numbers may contribute to statistical bias. Furthermore, the simulated keratometry and corneal topography examinations were not routinely performed for those who received DFCL and DIMS evaluations in our institution; thus, the analysis concerning the corneal curvature-related factor for myopic control cannot be executed. Finally, we excluded some individuals with DFCL and DIMS applications and astigmatism higher than −1.25 D, and a considerable number of individuals were excluded. Still, since the DFCL cannot correct the astigmatism, we decided to analyze the population with higher suitability rather than merely a large patient number.

In conclusion, the users of DIMS spectacle lens demonstrated similar SER and AXL control compared to the DFCL users, and the trend of AXL elongation was also similar between the DIMS group and the DFCL group, after adjusting several myopia-related parameters. Furthermore, patients with moderate myopia, higher AXL, and young age may benefit from DFCL and DIMS. Consequently, DIMS could be recommended for those with difficulty wearing contact lenses. Future prospective large-scale studies are mandatory to evaluate the efficiency of DFCL and DIMS spectacle lenses on high myopia control.

## Data Availability

The original contributions presented in the study are included in the article/supplementary material, further inquiries can be directed to the corresponding author.
